# Neuroborreliosis with Unusual Presentation: A Case Report

**DOI:** 10.7759/cureus.5758

**Published:** 2019-09-25

**Authors:** Salman Khan, Gurjaspreet K Bhattal, Nikhil H Shah, Jorge Lascano, Apurwa Karki

**Affiliations:** 1 Internal Medicine, Guthrie Clinic/Robert Packer Hospital, Sayre, USA; 2 Internal Medicine, University of Florida, Gainesville, USA; 3 Cardiology, University of Florida, Gainesville, USA; 4 Internal Medicine - Critical Care, Guthrie Clinic/Robert Packer Hospital, Sayre, USA

**Keywords:** neuroborreliosis, csf, lyme disease

## Abstract

Lyme disease is the most common vector-borne disease in the northern hemisphere. Neurological complications usually manifest in patients who do not receive treatment for Lyme disease. Neurological involvement may be early or late, depending on the duration of the symptoms. Early neuroborreliosis presents with symptoms such as headache and meningism; late neuroborreliosis can present with signs and symptoms of encephalopathy and stroke-like symptoms. The diagnosis is based on clinical manifestations and lumbar puncture finding. Treatment consists of intravenous antibiotics for a period of three to four weeks. Patients who receive early treatment usually have an excellent prognosis, with very few patients developing post-treatment Lyme disease syndrome. Here, we report an unusual case of Lyme disease with extremely high cerebrospinal fluid protein level and devastating neurological sequelae. The diagnosis of neuroborreliosis is based on neurological symptoms and lumbar puncture findings.

## Introduction

Lyme disease is a tick-borne illness caused by Borrelia burgdorferi, and it is the most common vector-borne disease in the northern hemisphere, characterized by the involvement of various organ systems [1]. Lyme disease involves the skin, joints, heart, and nervous system in different stages of the disease; the neurological involvement of Lyme disease is collectively termed neuroborreliosis [1]. Neuroborreliosis includes manifestations such as meningoradiculitis, lymphocytic meningitis, stroke-like symptoms, demyelinating disease, and, rarely, pseudotumor cerebri [1]. Neuroborreliosis may present as an early or late manifestation of Lyme disease; early presentation consists of a headache and meningism whereas late presentation is that of chronic meningitis, myelitis, encephalomyelitis, or vasculitis [2]. Here, we present a case of neuroborreliosis with very high cerebrospinal fluid (CSF) protein content and devastating neurological injury.

## Case presentation

A 41-year old female, with a past medical history of lupus, left-posterior parietal ventriculoperitoneal (VP) shunt due to congenital hydrocephalus, and migraine headaches, presented to our institution as a transfer from another facility for a two-month history of headaches, nausea, vomiting, 30-lb weight loss, and newly developed bilateral vision loss and dysphonia. At an outside hospital, her labs were significant for a creatinine (Cr) of 6.6 milligram/deciliter (normal 0.2-1.5). Computed tomography (CT) of the head was significant for over-shunting. She suffered a cardiac arrest on Day 2 of hospitalization, requiring defibrillation. Dopamine and vasopressin infusion was started for bradycardia and hypotension. Magnetic resonance imaging (MRI) of the brain reported an acute ischemic insult involving the cortex and subcortical white matter of the medial aspect of the left inferior parietal lobe adjacent to the VP shunt. She was then transferred to our facility for further management.

**Figure 1:**
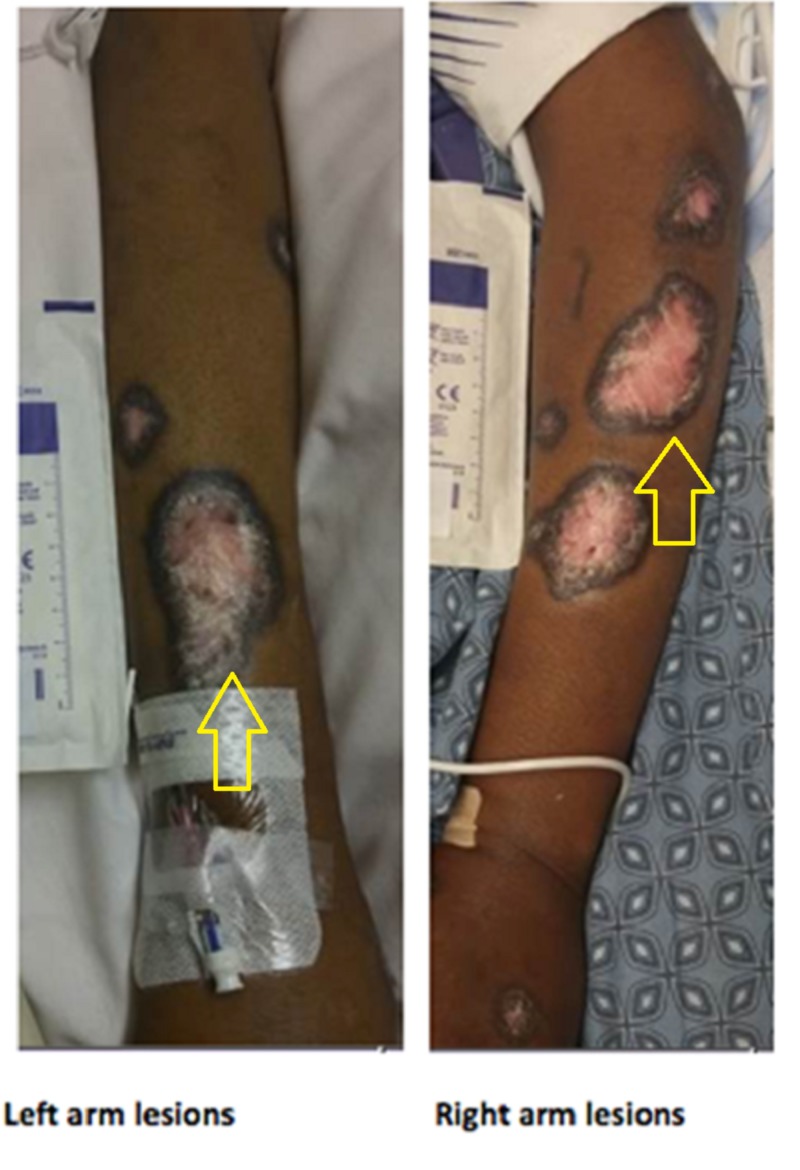
Left and Right Arm Lesions

The patient arrived at our facility intubated, sedated, requiring the infusion of norepinephrine and dopamine. Her physical exam was significant for sinus bradycardia, miotic pupils bilaterally, absent gag reflex, disconjugate gaze, and several well-demarcated, erythematous plaques on the extremities (Figure 1).

**Figure 2:**
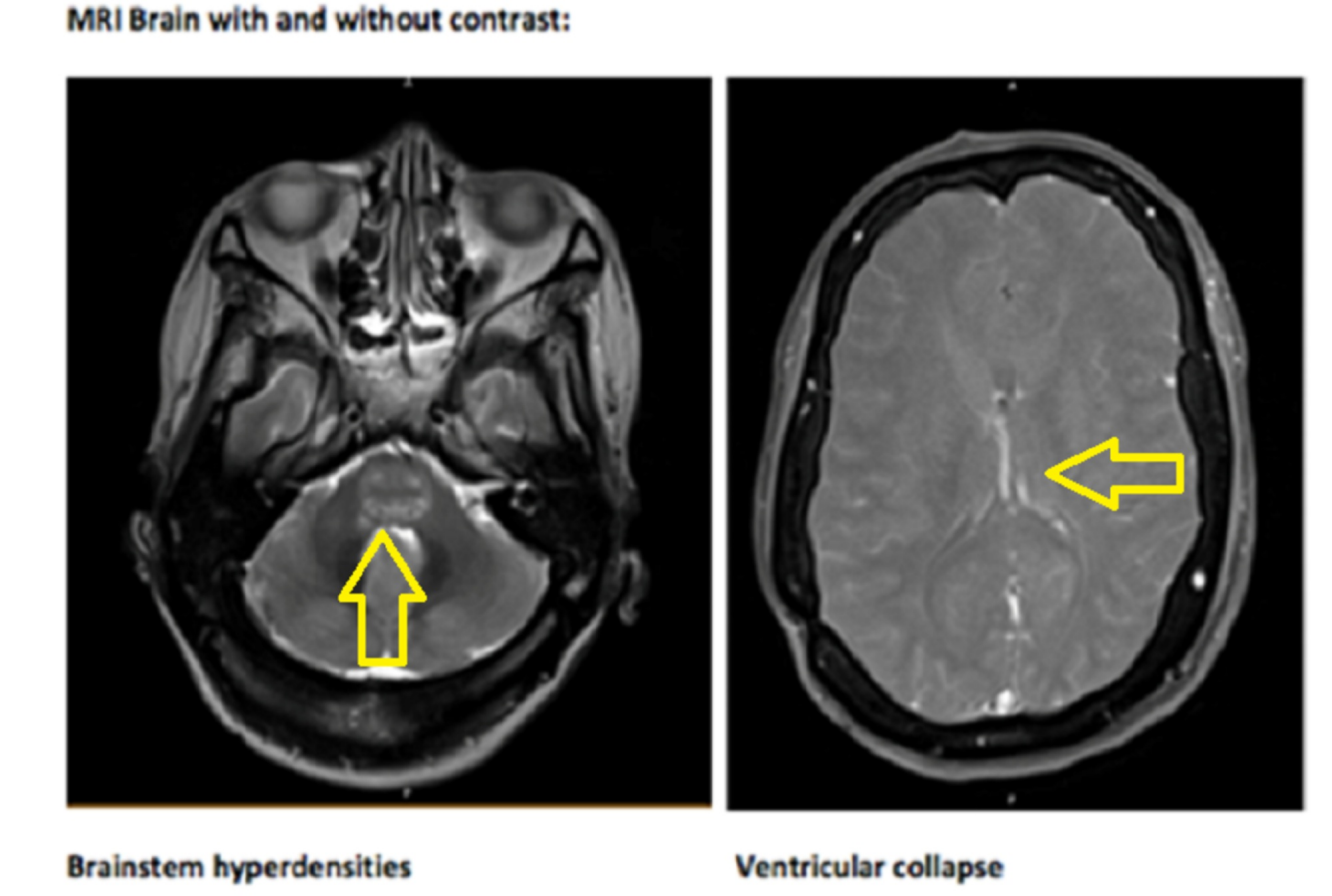
MRI Brain Showing brainstem hyperdensities and ventricular collapse

CT scan of the head was concerning for hypodensities in the diencephalon, bilateral mesiotemporal structures, and mesencephalon; no hydrocephalus was noted. A CT scan of the chest showed bibasilar pulmonary emboli and consolidation or possible areas of infarction in the lung. MRI brain revealed abnormal T2/FLAIR (fluid-attenuated inversion recovery) signal in the brainstem, periventricular white matter, right mesial temporal lobe, bilateral internal capsules, corpus callosum, and cerebellar vermis (Figure 2).

Rheumatology was consulted due to concerns of lupus cerebritis, lupus anti-coagulation, and lupus nephritis. However, normal levels of C3, C4, anticardiolipin antibody, and ds-ANA pointed towards discoid lupus rather than systemic lupus.

Slowly, the patient was weaned off of sedation and pressor support. Her heart rate (HR) remained around 60 beats/minute. Trans-thoracic echocardiogram revealed a left ventricular ejection fraction (EF) of 30%-35% with severely hypokinetic basal and mid-portions of all segments with relatively preserved apical segments. This pattern was thought to represent a variant of stress-induced cardiomyopathy. Blood and sputum urine cultures all remained negative.

A lumbar puncture performed on Day 3 of hospital admission showed white blood cells (WBCs) 318 cells/deciliter, red blood cells (RBCs) 466 cells/deciliter, polymorphonuclear cells (9%), lymphocytes 79%, monocyte 12%, protein of 1208 mg/dl (normal 15-45), slight xanthochromia, glucose 33 milligrams/deciliter (normal 40-70). Immunoglobulin G (IgG) was elevated at 235 milliliter/deciliter (normal 0-6 ml/dl), and oligoclonal bands were noted. Lumbar puncture results revealed elevated Borrelia (B.) burgdorferi antibodies in the cerebrospinal fluid (CSF) at 1.37 (normal <0.99) with elevated CSF IgG at 171 (normal 0 - 6.0 mg/dl). Her blood cultures remained negative as did serologic testing for B. burgdorferi. 

She was started on intravenous (IV) ceftriaxone for a 28-day course, however, she remained vent-dependent, with minimal improvement in her overall condition. A goals-of-care discussion was held, and the family decided to withdraw care after a two-week hospital stay. She passed away soon after relieving her from the ventilator with her family at her bedside.

## Discussion

The normal CSF protein content is 15-45 milligram/deciliter [3]. Infections could cause an elevation of CSF protein content, however, an isolated elevation of protein level with relative normalcy of other measured parameters is uncommon. Bacterial meningitis causing CSF protein levels to be more than 1000 mg/dl has been reported but is uncommon in the presence of negative gram stain and CSF pleocytosis [4]. The major causes of very high CSF protein are blood in the CSF, Froin’s syndrome, and inflammatory and demyelinating conditions, including multiple sclerosis, neurosyphilis, subacute sclerosing panencephalitis, viral encephalitis, and sarcoidosis [5-6]. Froin’s syndrome is the presence of spinal canal blockage by tumor or infection that causes the loculation of CSF and can result in a very high CSF protein content [6]. The primary pathophysiology of such a degree of protein content in CSF in the conditions mentioned above is thought to be the secretion of IgG due to various underlying conditions [5,7]. The diagnosis of each condition is based on the clinical features as well as laboratory investigations.

Lyme disease is a reportable disease in the United States since 1991 and is endemic in certain states in the country, including the Northeast, Mid-Atlantic, upper Mid-West, and certain areas of the Pacific coast [8]. The manifestations of Lyme disease can vary depending on the stage of the disease, and it has been postulated that with appropriate antibiotic treatment, patients usually experience full recovery [9].

As discussed earlier, Lyme disease-related neurological symptoms may present early or late in the course of the disease. The diagnosis of neuroborreliosis is based on neurological symptoms and lumbar puncture findings [1]. The clinical features of early neuroborreliosis include lymphocytic meningitis, cranial neuritis with unilateral or bilateral facial nerve palsy, and intermittent attacks of severe headaches [10]. Late neuroborreliosis is the presence of continuous disease activity lasting more than six months and the symptoms are characterized by chronic meningitis, progressive encephalitis, myelitis or encephalomyelitis, and cerebral vasculitis [1,11]. Cerebral vasculitis and stroke-like symptoms are reported in the young without any underlying risk factors for stroke; these patients usually have worse long-term outcomes as compared to typical patients with stroke [12].

Two entities are often described in relation to Lyme disease: post-treatment Lyme disease syndrome (PTLDS) and chronic Lyme disease (CLD). PTLDS is a congregation of subjective symptoms: fatigue, arthralgia, myalgia, and perceived cognitive impairment that persists for at least six months after conventional treatment [1]. The Infectious Disease Society of America has proposed diagnostic criteria for PTLDS that include objective proof of previous Lyme disease, the presence of subjective symptoms that compromise function in daily life, and a lack of evidence of another underlying illness that could explain the symptom [13]. There is a lack of compelling evidence that the syndrome is secondary to an ongoing infection and the treatment should be directed towards the symptoms [1]. CLD has been used in the past to describe patients with poorly defined symptoms who may or may not have evidence of exposure and/or Lyme disease in the past [14]. Patients with no history of Lyme disease but only serological evidence of exposure or with some other disease process that would give rise to the symptoms of Lyme disease are some of the patient groups identified as having CLD, and the very existence of this condition is in question, as there is very little evidence to support its diagnosis [1,14].

The diagnosis of Lyme disease is based on the history of exposure, although 50%-70% of patients do not recall a tick bite, clinical manifestation, or laboratory criteria [15]. A two-tier serology test for the confirmation of Lyme disease has been approved by the Centers for Disease Control (CDC) in case the other criteria are not met, although the test may be negative within four to six weeks of the tick bite, which might be the period for seroconversion [16]. The two-tier test consists of the initial highly sensitive enzyme-linked immunosorbent assay (ELISA) test followed by a more specific Western blot test [16]. The serological test is also reported to be falsely negative in patients with neuroborreliosis and underlying immunosuppression [17]. There is an algorithm proposed by Koedel et al., which bases the diagnosis of neuroborreliosis on the presence of symptoms, followed by lymphocytic pleocytosis in CSF, and, finally, evidence of either B. burgdorferi antibody, polymerase chain reaction (PCR), or chemokine CXCL13 in the CSF [1]. Other diagnostic tools include the use of the antigen test of the body fluid, PCR of urine samples, lymphocyte transformation test, enzyme-linked immunospot assays, CD57 natural killer count, and xenodiagnosis [18]. As these tests are not validated scientifically for Lyme neuroborreliosis, their use for routine diagnosis cannot be recommended [1]. The use of chemokine CXCL13 has been studied as a biomarker with high sensitivity and specificity and has been shown to be present before the B. burgdorferi antibody [1,19]. CXCL3 is elevated in CSF in cases of primary and secondary CSF lymphoma, tuberculous meningitis, and neurosyphilis, and these conditions should be ruled out before considering Lyme neuroborreliosis [20]. So, this biomarker could be helpful in patients with early neuroborreliosis with typical symptoms and negative CSF B. burgdorferi antibodies [1].

## Conclusions

Neuroborreliosis is a neurological condition that can be diagnosed with the help of lumbar puncture findings. Along with the presence of symptoms, the CSF findings are consistent with lymphocytic pleocytosis. Although not a standard of care, the use of chemokines can be beneficial in early detection.

## References

[1] Koedel U, Fingerle V, Pfister H (2015). Lyme neuroborreliosis—epidemiology, diagnosis and management. Nat Rev Neurol.

[2] Topakian R, Stieglbauer K, Aichner F (2007). Unexplained cerebral vasculitis and stroke: keep Lyme neuroborreliosis in mind. Lancet Neurol.

[3] Dunbar S, Eason R, Musher D, Clarridge J (1998). Microscopic examination and broth culture of cerebrospinal fluid in diagnosis of meningitis. J Clin Microbiol.

[4] Spanos A, Harrell F, Durack D (1989). Differential diagnosis of acute meningitis. JAMA.

[5] Emory University School of Medicine, Atlanta Atlanta, Georgia Georgia (1990). Clinical Methods: The History, Physical, and Laboratory Examinations. https://www.ncbi.nlm.nih.gov/pubmed/21250045.

[6] Mirza S, Adams W, Corkhill R (2008). Froin's syndrome revisited, 100 years on. Pseudo-Froin's syndrome on MRI. Clin Radiol.

[7] Baig S, Olsson T, Hojeberg B, Link H (1991). Cells secreting antibodies to myelin basic protein in cerebrospinal fluid of patients with Lyme neuroborreliosis. Neurology.

[8] Schwartz A, Hinckley A, Mead P, Hook S, Kugeler K (2017). Surveillance for Lyme disease—united states, 2008-2015. MMWR Surveill Summ.

[9] Hu L (2016). Lyme disease. Ann Intern Med.

[10] Reik L, Steere A, Bartenhagen N, Shope R, Malawista S (1979). Neurologic abnormalities of Lyme disease. Medicine.

[11] Hansen K, Lebech A (1992). The clinical and epidemiological profile of Lyme neuroborreliosis in Denmark 1985-1990. A prospective study of 187 patients with Borrelia burgdorferi specific intrathecal antibody production. Brain.

[12] Lebas A, Toulgoat F, Saliou G, Husson B, Tardieu M (2012). Stroke due to Lyme neuroborreliosis: changes in vessel wall contrast enhancement. J Neuroimaging.

[13] Wormser G, Dattwyler R, Shapiro E (2006). The clinical assessment, treatment, and prevention of Lyme disease, human granulocytic anaplasmosis, and babesiosis: clinical practice guidelines by the Infectious Diseases Society of America. Clin Infect Dis.

[14] Feder H, Johnson B, O'Connell S, Shapiro E, Steere A, Wormser GP, Ad Hoc International Lyme Disease Group (2007). A critical appraisal of “chronic Lyme disease”. New Engl J Med.

[15] Bratton R, Whiteside J, Hovan M, Engle R, Edwards F (2008). Diagnosis and treatment of Lyme disease. Mayo Clin Proc.

[16] Harrer T, Geissdorfer W, Schoerner C, Lang E, Helm G (2007). Seronegative Lyme neuroborreliosis in a patient on treatment for chronic lymphatic leukemia. Infection.

[17] Rupprecht T, Lechner C, Tumani H, Fingerle V (2014). CXCL13: a biomarker for acute Lyme neuroborreliosis: investigation of the predictive value in the clinical routine [Article in German]. Der Nervenarzt.

[18] van Dop W, Kersten M, de Weaver B, Hovius J (2013). Seronegative Lyme neuroborreliosis in a patient using rituximab. BMJ Case Rep.

[19] Schmidt C, Plate A, Angele B, Pfister H, Wick M, Koedel U, Rupprecht TA (2011). A prospective study on the role of CXCL13 in Lyme neuroborreliosis. Neurology.

[20] Rubenstein J, Wong V, Kadoch C (2013). CXCL13 plus interleukin 10 is highly specific for the diagnosis of CNS lymphoma. Blood.

